# Neurochemical Changes in the Aging Process: Implications in Medication in the Elderly

**DOI:** 10.1100/tsw.2007.112

**Published:** 2007-09-28

**Authors:** Ebere C. Anyanwu

**Affiliations:** St. Peter's Middle College House, 190 College Road, Birmingham, UK

**Keywords:** Aging process, chemical changes, health effects, elderly medication

## Abstract

Aging is an inevitable process in human development, which follows a time course thatcan be delayed, or hastened, by lifestyles, diseases and events. The factors that affectthe aging process can be delayed, but not prevented. This paper evaluates theneurochemical changes in the aging process and their relevance in the modality ofelderly medication. For clarity and understanding of the relevant neurobiochemicalprocess and effects, the neuroanatomical, physiological, and neurobehavioral changesare reviewed as they relate to medication in the elderly.

## INTRODUCTION

The aging process is an inevitable consequence of developmental process in life, that is, there is no aging without life and vice versa. The process can be delayed or hastened, depending on individual lifestyles, diseases, and environmental events. However, it is the temporal and quality of the process of aging that determines normal longevity. Normal aging is associated with a series of structural, functional, and biochemical alterations that could hinder or promote the process. Consequently, there is a decrease in the number and size of neurons; loss of synapses and neuronal branching with reduced functioning of neurotransmitter systems, such as the serotonergic system[1]. These structural, functional, and biochemical alterations have important impacts on the behavioral, cognitive, and affective status of the individual.

In the last 25 years, there have been rigorous efforts by clinicians and researchers to unravel and understand the molecular and cellular basis of the aging brain and how they relate to quality of life. Aging is associated with a decline in cognitive performance, and several neurological disease states, such as Alzheimer's disease (AD), multiple sclerosis (MS), corpora amylacea, argyrophilic grains, neuromelanin, and lipofuscin. Both normal aging and neurological disease states are associated with an overlapping increase in the levels of pathology, which are potential mediators of cognitive decline in the elderly[1].

The aim of this review is to evaluate the neurochemical changes in aging, which may hasten or delay the process and the implications in elderly medication. Relevant peer reviewed literatures from the major databases especially Medline, Pubmed, EMBASE, Expert reports and discussions are searched to provide in-depth information about the factors that affect aging process as they relate to elderly medication, using the following search criteria:

i. Neuroanatomical changes in aging process ii. Neurophysiological changes in aging process
iii. Biochemical changes in aging process
iv. Neurobehavioral changes in aging process
v. Cognitive changes in aging
vi. Medication in aging

## Neuroanatomical Changes

Aging is often accompanied by learning and memory problems, many of which resemble deficits associated with hippocampal damage. Although the hippocampus undergoes structural and biochemical changes with normal aging process, these changes may represent an important component of age-related deterioration in hippocampus-dependent cognition[2]. The effects are regionally distributed in the brain especially in the regions of the neocortex and the hippocampal dentate gyrus. This decline in brain status occurs early in the disease process and strongly correlates with the cognitive decline observed in AD. The synapse loss does not appear to be an inevitable consequence of the aging process[3]. However, a distinct subpopulation of neurons forming long corticocortical projections in the association neocortex is highly vulnerable to the degenerative process in AD. Hence, cortical neurons furnishing long and short corticocortical projections display consistent neurochemical changes during aging to which a differential decrease in cellular expression of glutamate receptor subunit occurs[4]. The aging process produces morphological and neurochemical alterations in the suprachiasmatic nuclei. Forebrain areas, such as the caudate-putamen complex, globus pallidus, prefrontal and frontoparietal cortices are consistently affected, whereas serotonergic fibers are only infrequently affected in other areas like septal and amygdaloid nuclei[5].

## Changes in Brain-Blood Barrier

One possible cause of the decline in brain function with aging might be an age-related change in the blood-brain barrier (BBB). Therefore, the status of the intracellular activity of biomolecular compounds such as methionine, lysine, glutamic acid and N-methyl-N-nitrosourea (MNU) plays important roles in the aging process. The BBB undergoes different changes in development and aging, leading to an optimal amino acid transport and minimum penetration of lipophilic agents in the adult brain, reducing necessary transport and increasing the penetration of lipophilic compounds[6].

In some research findings, regional decrease of cerebral blood-flow is noted in the fronto- and temporocortical area and in the subcortical region of the brain. Aging process is negatively correlated with perfusion in the left and right fronto-cortical region. The binding index of the neuroreceptor radioligand is decreased in the fronto-cortical region, with a significant negative correlation with age in the right fronto-cortical area [7].

Blood-to-brain amino acid transport consists of at least two components, a fast rate or early process, commonly measured by the intra-carotid bolus injection method and attributed to transport across the capillary endothelium and entry into the astrocytes, and a slow rate or later component measured over 2 to 15 minutes probably associated with exit from the astrocytes and entry into the neurons[8]. The fast rate transport component is unaffected by age while the rates of the slow process and protein turnover show an exponential decline. These changes suggest that brain protein metabolism is altered qualitatively as well as quantitatively in aging process[8].

## Neurochemical Changes

Neurochemical data similarly revealed regional differences for example, 5-Hydroxytryptamine levels are increased in the frontoparietal cortex, hippocampus, hypothalamus, and the mesencephalic raphe region but remained unchanged in the caudate-putamen complex[5]. Loss of dopamine synthesis in the striatum with normal human aging has been observed in the postmortem brain. Significant age-related decline of dopamine synthesis is observed in the striatum and extrastriatal regions except midbrain. The decline in the overall uptake rate constant is more prominent than in the tissue fraction of gray matter. Thus, indicating that age-related decline in striatal dopamine synthesis extends to several extrastriatal regions in normal human aging brain[9].

The striatum is particularly vulnerable to oxidative stress produced by sodium azide and produces striatal lesions by a secondary excitotoxic mechanism[10]. Most of the neurological diseases associated with aging for example, Alzheimer's disease are characterized by the accumulation of neuropathologic lesions and neurochemical alterations. There is significant evidence for age-related declines in the number of N-methyl-D-aspartate (NMDA) receptors in the CA1, CA3 and subicular cell regions of the hippocampus and in the number of alpha-amino-3-hydroxy-5-methyl-4-isoxazole (AMPA) receptors. However, this decline in the number of NMDA or AMPA receptors is not a continuous or homogeneous process[11].

Decline in vision is one of the early problems of aging. Neurochemical changes are evident before degeneration, involving the amino acids glutamate, GABA, aspartate, glutamine, and arginine. Subsequently, these changes that become evident during photoreceptor degeneration involve the amino acids taurine and glycine. Anomalies in uptake characteristics are also evident during the degeneration phase and are likely to reflect changes in cellular function because of the degeneration process. Neurochemical changes evident before photoreceptor degeneration involve both glutamate and GABA manufacturing pathways[12].

Aging process shows a progressive failure of the pineal gland’s capacity to secrete nighttime plasma melatonin. The decline in melatonin secretion with age occurs at an average rate of 10-15% per decade. This is used as a marker in human brain aging and to estimate plasma melatonin levels in normal aging and pathological age-related changes. In Alzheimer disease for example, melatonin secretion is significantly lower compared to age matched normal subjects[13].

Compared with healthy women, frail women had higher rates of whole-body protein synthesis and breakdown per kg fat-free mass and lower rates of muscle protein breakdown when expressed as total amounts per day but higher rates when expressed per kg muscle[14]. The protein-enriched diet had no effect on these variables but resulted in an increase in net endogenous protein balance and a positive nitrogen balance at the end of the diet period. Frailty exacerbates age-related changes in protein metabolism by inducing an increase in muscle protein catabolism and a decrease in muscle mass. At low protein intakes, the increase in muscle catabolism may be a form of protection for both nonmuscle lean tissue mass and function at the expense of muscle mass. Frail women maintained the capacity to retain nitrogen after increased protein intakes, which could convey health benefits if sustained over a long enough period to result in lean tissue accretion[14].

## Decline in Neurotransmitters and Enzyme Activities

Neurochemical and histochemical techniques for characterization of neurotransmitters and their receptors in normal and pathological human brain showed three types of neuromediator or modulator. These are: cholinergic, aminergic and peptidergic, that appear to be affected to different degrees in five electively involved anatomical systems: cholinergic innominocorticoamygdalian and septohippocampic systems, noradrenergic ceruleocortical system, serotoninergic pontocortical system and cortical somatostatin and substance P systems[15]. Significant alterations with increasing age (from 61 to 92 years) in a series of non-demented cases included a reduction of the cholinergic enzyme, choline acetyltransferase, and an increase in vasoactive intestinal peptide immunoreactivity[16].

Furthermore, alterations in other neurochemical activities such as reductions in dopamine-beta-hydroxylase activity, cholecystokinin octapeptide and somatostatin immunoreactivities and an increase in substance P immunoreactivity may occur at later stages of disease process[16]. The levels of neurotransmitters (Acetylcholine (ACh), serotonin (5-HT) and dopamine) levels all decreased with age but this decrease and related metabolic enzyme activities such as choline acetyltransferase and choline esterase activities in the brain increase with age. Tryptophan hydroxylase activity is not affected by age. However, monoamine oxidase-A activity increased with age[17].

## Adverse Neurobehavioral Changes

Structures of the medial temporal lobes are recognized to play a central role in memory processing and to be the primary sites of deterioration in AD. Mild cognitive impairment (MCI) represents potentially an intermediate state between normal aging and AD. Patients with AD and MCI demonstrated the same metabolic pattern suggesting a similar pathological process underlying memory impairment. Some interhemispheric metabolite asymmetries were increased in AD patients[18]. These neurochemical changes are accompanied by changes of motor behavior, the main neurobiological process regulated by nigrostriatal dopaminergic neurons; neurochemical indices--dopamine (DA) and L-3,4-dihydroxyphenylacetic acid (DOPAC) contents, tyrosine hydroxylase (TH) activity, and number and affinity of D1 and D2 dopaminergic receptors in the striatum--and behavioral parameters--spontaneous locomotor activity and spontaneous and induced stereotypic behavior--both indicating nigrostriatal dopaminergic activity, in adult. There was a significant increase in the DOPAC/DA ratio[19]. Aging process shows major alterations in the quality of sleep and frequently accompanied by changes in life style due to different, often less demanding, social and occupational activities, leading to an attenuation of the synchronizing effects of the light-dark and activity-rest cycles[20].

The concentrations of monoamines and cholineacetyl transferase (CAT) are reduced in brains from patients with normal aging. According to findings about the DA system, the age-sensitive decrease has its onset in the 60s and after that has a continuous progress[21]. The activity of monoamine oxidase B (MAO-B) increases significantly with age. Increased enzyme activity is considered a marker of gliosis in the brain and a marker of macrocytosis in platelets. Gangliosides are reduced in several areas of the brain, which indicates a reduction of nerve terminals. Significantly reduced concentrations of myelin components have also been recorded in brains, which is in agreement with histopathological findings[21]. The loss of tolerance accompanying aging may be indicative of more widespread changes in CNS adaptability in this strain. The unequal capacity to adapt also appears to depend on the nervous structure and the neurotransmitter considered. Neurochemical effect might depend on the nervous structure or neurotransmitter involved. Such a phenomenon may be the basis of differences in behavioral changes observed in alcoholics[22].

## Age-Related Decline in Brain Nutritional Factor

A possible role of the macronutrients and the basic elements of carbohydrates (glucose administration or depletion), proteins (amino acids such as tryptophan and tyrosine), and fat (unsaturated fatty acids) was recently proposed for age-related changes of cognitive function, and the cognitive decline of degenerative (AD) or vascular origin[23]. The availability and utilization of glucose has been implicated in cognitive function not only as a result of nutritional and systemic metabolic conditions, but also, as a crucial phase of the mechanism of action of molecules used as cognitive-enhancers. Furthermore, many lines of evidence have focused on the importance of oxidative stress mechanisms and free radical damage in AD pathogenesis[23].

Homocysteine, a sulfur-containing amino acid, is a metabolite of the essential amino acid methionine, and exists at a critical biochemical intersection in the methionine cycle between S-adenosylmethionine, the indispensable ubiquitous methyl donor, and vitamins B12 and folic acid. High blood levels of homocysteine signal a breakdown in this vital process, resulting in far-reaching biochemical and life consequences[24,25]. The link between homocysteine and cardiovascular disease is well established, and decreasing plasma total homocysteine by providing nutritional cofactors for its metabolism has been shown to reduce the risk of cardiovascular events. Information has been emerging regarding a connection between homocysteine metabolism and cognitive function, from mild cognitive decline (age-related memory loss) to vascular dementia and Alzheimer's disease.

## Age-related Decline in Brain Ion

Brain performances, e.g. learning and memory, decay during aging. Deterioration of synaptic junctions, as structural correlates of these key functions of the central nervous system, may play a central role in this impairment. Brain numeric loss of synaptic contacts and size of overall synaptic junctional area per cubic micron of neuropil is also reduced in aging, which account for the age-associated functional decay of CNS performances[26]. There is a progressive decline of the mitochondrial metabolic competence, i.e. the capacity of select pools of organelles to provide adequate amounts of adenosine triphosphate. There is a loss of key enzymes of the respiratory chain involving cytochrome oxidase and succinic dehydrogenase[26].

The compensatory mechanisms such as additional prefrontal cortical activity are called upon to maintain proficiency in task performance. As cognitive demand increases, however, they are pushed past a threshold beyond which physiological compensation cannot be made and, a decline in performance occurs[27]. Although aluminium (Al) has been implicated in various neuropathological states with aging due to its involvement in neurotoxicity by inhibiting catalase activity but this effect may not be a major contributing factor in the aging of the brain if antioxidant capacity is maintained in old age[28]. Nitric oxide (NO) has been implicated in the mediation of the neuronal excitotoxic cascade. It is suggested that an increase in NO production could be due to the concomitant neuronal damage rather than seizure activity per se[29]. A decline in the ability to discriminate speech from noise due to age-related hearing loss (presbycusis) may reflect impaired auditory information processing within the central nervous system probably due to functional loss of the inhibitory neurotransmitter GABA. Age-related change in GABA(A) receptor composition. These changes may reflect a compensatory up-regulation of inhibitory function in the face of significant loss of presynaptic GABA release. This is an example of plastic neurotransmitter receptor changes, which can occur during the aging process[30].

## Medication in the Elderly

Medication in the elderly is an emerging source of age-related metabolic dysfunctions to which unusual and unhealthy rapid increases in weight gains abound. Many elderly people are often prescribed several structurally and functionally identical painkillers for example, which exacerbate the levels of pain with excruciating severity. There is a consistent tendency for the elderly to be given polypharmacotherapy that could interact and cause health conditions that were different from the initial complaint of the patient. Intracellular drug interactions and the age-related changes discussed thus far should be considered in elderly medication. It becomes evident that the synergistic painkiller interactions with the cellular metabolites for example, could have the propensity to cause more harm than good. Consequently, on contact, the antagonists of the NMDA glutamate receptors will create an intracellular cascade of neurophysiological and behavioral entropy resulting for example, to cognitive and behavioral changes in the elderly. This scenario could be one of the reasons for recent frequent newspaper and radio reports of amnesia among the elderly.

Several lines of evidence suggest that a large number of these painkiller drug-induced effects are dose-dependent manifestations of the same general disinhibition process in which NMDA antagonists abolish GABAergic inhibition, resulting in the simultaneous excessive release of acetylcholine and glutamate. Progressive increases in the severity of NMDA receptor hypofunction within the brain thereby increasing a range of effects on brain function[31]. In addition, underexcitation of NMDA receptors by relatively low doses of NMDA antagonist drugs can produce specific forms of memory dysfunction without evident psychosis[31]. It is necessary to consider the possible adverse effects of the drug functional group interactions when putting an elderly under certain medications. This will in effect, minimize some of the degenerative processes that were not there in the first place.

## Importance of B12, B6 and Folate

Significant deficiencies in the homocysteine re-methylation cofactors, cobalamin (B12) and folate, as well as the trans-sulfuration cofactor vitamin B6, are commonly seen in the elderly population, with a resultant increase in homocysteine with advancing age. Hyperhomocysteinemia has been shown to be an independent risk factor for cognitive dysfunction. Indirect and direct vascular damage can be caused by homocysteine, which has been implicated in vascular dementia, with an increased risk of multiple brain infarcts and dementia as homocysteine levels rise (24,32). A significant correlation has been found between risk of Alzheimer's disease and high plasma levels of homocysteine, as well as low levels of folic acid, and vitamins B6 and B12. All of these disease associations are thought to be interrelated via increased homocysteine and S-adenosylhomocysteine and subsequent hypomethylation of numerous substances, including DNA and proteins, that render vascular structures and neurons more susceptible to damage and apoptosis. Providing the nutritional cofactors for proper functioning of the methionine cycle may improve methylation and protect the brain from damage.

Further studies need to be performed to assess whether this will also reduce the risk of cognitive diseases and/or improve cognitive functioning[24,32]. The age-associated immunosuppression may be inversely related to the hypothalamic glutamatergic activity and consumption of diets having variable quantity of protein without variation of calorie content modulates immune response and hypothalamic glutamatergic activity depending upon age and duration of dietary supplementation[33]. Aging is associated with a decline in motor coordination and the ability to learn new motor learning skills and is correlated with a decline in cerebellar beta-adrenergic receptor function. Exogenous oxidative insult results in a decline in cerebellar beta-adrenergic receptor function. The effect of hyperoxia is blocked by antioxidants. Therefore, foods such as blueberries and spinach can prevent and/or reverse age-related declines in cerebellar noradrenergic receptor function[25]. Taurine is present in high concentration in the mammalian brain and is known to decline with aging[32,34].

## CONCLUSIONS

The neurochemical changes in aging process and the implications and relevance in elderly medication have been evaluated and discussed. It an accepted fact that aging is an inevitable process in human development, which follows a time course that can be delayed, or hastened, by lifestyles, diseases, and events. It recommended that the metabolic status of the elderly should be determined for appropriate choice of drug. In terms of painkiller drug prescription and intracellular interactions, it is important to observe caution not to administer drugs that are functionally and mechanistically identical, though they may be in different generic and chemical names. This will assist in avoiding unnecessary drug overdose and consequently, synergistic metabolic dysfunctions.
